# Grade of retraction and tendon thickness correlates with MR-spectroscopically measured amount of fatty degeneration in full thickness supraspinatus tears

**DOI:** 10.1186/s12891-018-2096-5

**Published:** 2018-06-21

**Authors:** F. Gilbert, R. H. Meffert, J. Schmalzl, A. M. Weng, H. Köstler, L. Eden

**Affiliations:** 1 Department of Trauma, Hand, Plastic and Reconstructive Surgery, Julius-Maximilians-University of Würzburg, Oberdürrbacherstr. 6, D-97080 Würzburg, Germany; 2 Department of Traumatology and Hand Surgery, St. Vincentius Klinik, ViDia Kliniken, Suedendstraße 32, D-76137 Karlsruhe, Germany; 3 Department of Radiology, Julius-Maximilians-University of Würzburg, Oberdürrbacherstr. 6, D-97080 Würzburg, Germany

**Keywords:** Rotator cuff, MRI, Spectroscopy, Muscle degeneration

## Abstract

**Background:**

The amount of fatty degeneration (FD) has major impact on the clinical result and cuff integrity after rotator cuff repair. A quantitative analysis with magnet resonance imaging (MRI) spectroscopy was employed to analyze possible correlation of FD with tendon retraction, tendon thickness and patients’ characteristics in full thickness supraspinatus tears.

**Methods:**

Forty-two patients with full-thickness supraspinatus tears underwent shoulder MRI including an experimental spectroscopic sequence allowing quantification of the fat fraction in the supraspinatus muscle belly. The amount of fatty degeneration was correlated with tendon retraction, tendon thickness, patients’ age, gender, smoker status, symptom duration and body mass index (BMI). Patients were divided in to three groups of retraction (A) 0-10 mm (*n*=), (B) 11-20 mm (*n*=) and (C) < 21 mm (*n*=) and the means of FD for each group were calculated.

**Results:**

Tendon retraction (*R* = 0.6) and symptom duration (R = 0.6) correlated positively, whereas tendon thickness correlated negatively (*R* = − 0.6) with the amount of FD. The fat fraction increased significantly with tendon retraction: Group (A) showed a mean fat mount of 3.7% (±4%), group (B) of 16.7% (±8.2%) and group (C) of 37.5% (±19%). BMI, age and smoker-status only showed weak to moderate correlation with the amount of FD in this cohort.

**Conclusion:**

MRI spectroscopy revealed significantly higher amount of fat with increasing grade of retraction, symptom duration and decreased tendon thickness. Thus, these parameters may indirectly be associated with the severity of tendon disease.

## Background

Fatty degeneration (FD) of the rotator cuff (RC) muscles is a major influencing factor for the anatomical and the clinical result after RC repair, with significant FD leading to inferior clinical results and showing higher failure rates of RC repair [1, 2]. Investigation of FD is thus essential in preoperative planning of RC repair [3–6]. The clinically widely used Goutallier scale represents a five-point semi-quantitative grading of the amount of FD and shows only low to moderate inter-observer reliability and low correlation compared to quantitative analyses [7–11]. As FD is irreversible even after successful RC repair, the surgeon has to rely on early detection and a reliable quantification of the fat fraction [12, 13]. Semi-quantitative studies using the Goutallier scale indicate a correlation between tear size, age, retraction and number of tendons involved. Several efforts to quantify the fat fraction, as single voxel spectroscopy, MR spectroscopy and chemical shift-based water-fat separation have been made. These methods have demonstrated a strong correlation of tear size and amount of FD in heterogeneous cohorts [14–16]. Nevertheless quantitative methods are lacking methodical standardization and may be difficult to apply in a clinical setting, thus these techniques inevitably remain experimental and the semi-quantitative Goutallier-scale is still regarded as the “gold standard”. MR Spectroscopy has been shown to enable accurate and reliable quantification of fat tissue in the RC and has been tested with other quantitative methods [8, 15, 17, 18]. The possible integration into a clinical MR scanner and the relatively easy post-processing make it attractive for clinical investigations regarding fat quantification and may add value and accuracy to evaluate the context of FD in RC tears [17].

The SPLASH technique (spectroscopic fast low angle shot) as an MR based spectroscopy allows quantification of the emitted tissue-specific spectrum in a 2nd MRI layer, its reproducibility and reliability has been reported previously [8, 15, 17]. This study investigates whether the amount of FD correlates with tendon retraction and tendon thickness and patient’s baseline characteristics as age, sex, BMI, smoker status and symptom duration when MR spectroscopy is used for quantification in full thickness supraspinatus tears.

## Methods

**Fig. 1:**
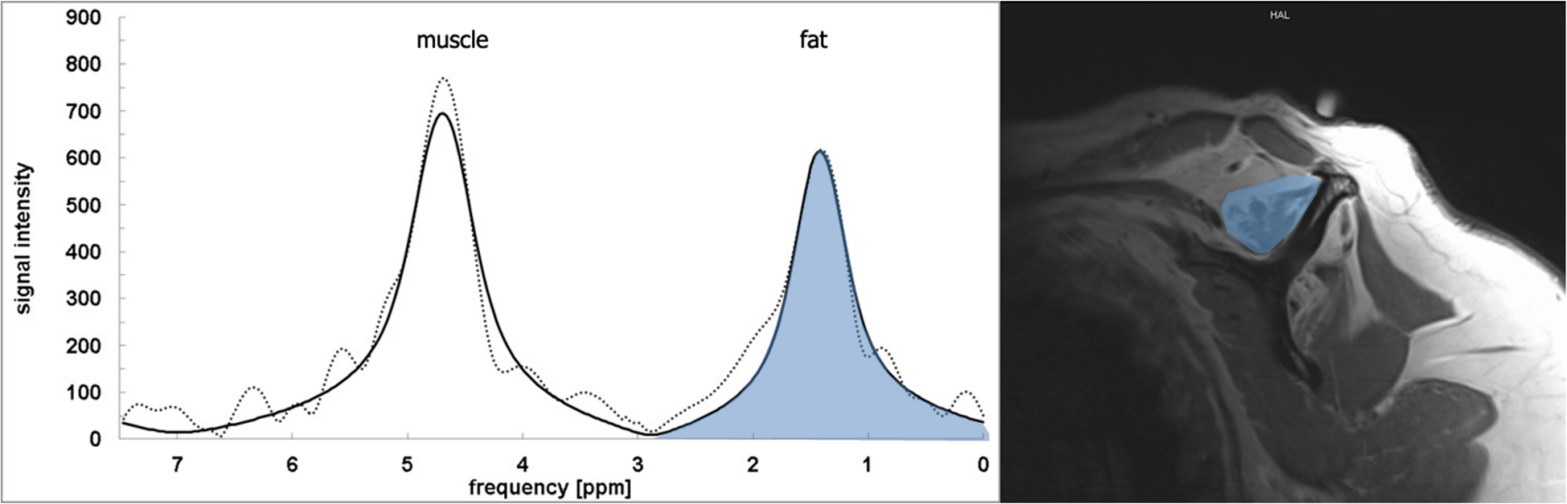
MRI Spectroscopy and para sagittal MRI of the Fossa supraspinata: MRI spectroscopy revealed a fat fraction of 44%. The amount of fat is represented as the area under the curve from the second peak in the spectrogram, the first peak represents water respectively muscle

Forty-Two Patients with full thickness supraspinatus tears were included in the study. A regular MRI shoulder image was performed, which included a spectroscopy using the SPLASH (spectroscopic fast low angle shot) technique. The exact technical background, post processing and calculation of the fat fraction have been previously described by our workgroup [8, 17]. MR-Spectroscopy allows quantification in a random region of interest; for all scans a 3 Tesla MRI (Skyra, Siemens, Germany) was used. MRI parameters for the t1-weighted images were: TR = 653 ms, TE = 12 ms, FOV 180 mm and for the SPLASH Sequence: TR = 35 ms, TE = 5-25 ms, FOV 278 mm (TR = repetition time, TE = echo time, FOV = field of view). Slices were 5 mm for the spectroscopy (SPLASH technique) and 3 mm for the standard MRI. For spectroscopic analysis, the muscular borders of the supraspinatus were delineated manually, and the quantitative evaluation of the spectra was obtained using a home-built reconstruction program (MATLAB 2014b, The MathWorks, Inc., Natick, Massachusetts, United States) and the time domain fit program AMARES implemented in jMRUI) (Fig. 1).

Patient’s demographic data as sex, age, smoker status, BMI and the onset of symptoms were collected from our clinical database.

**Fig. 2:**
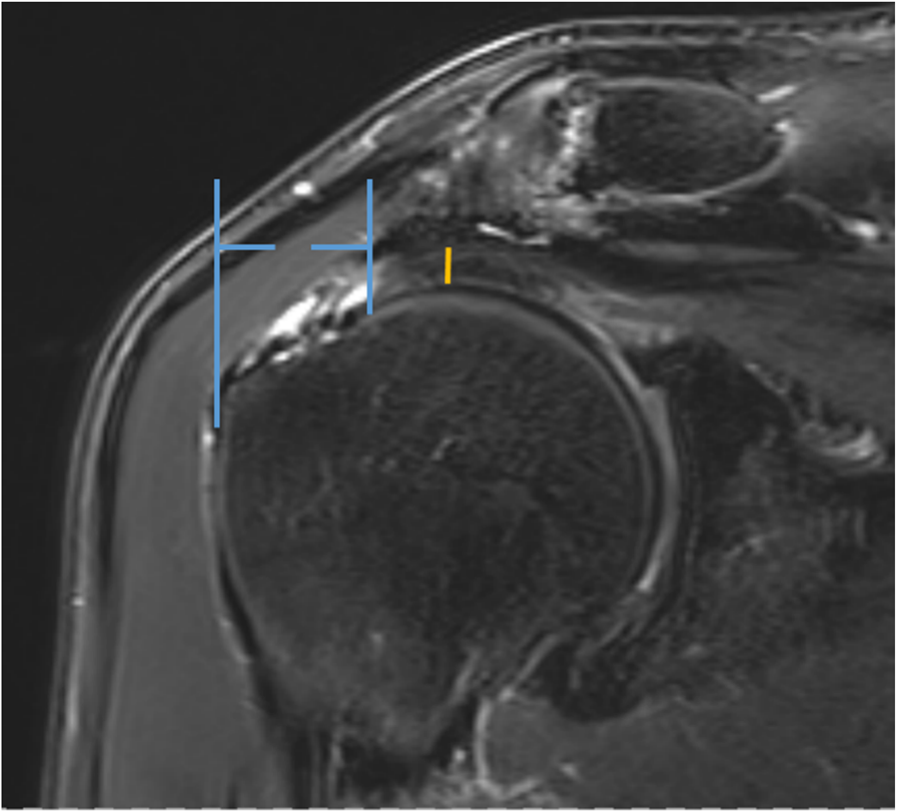
Tendon retraction and thickness were measured in the coronar plane of a t2-weighted MRI slice. Tendon retraction was measured between the lateral margin of the ruptured tendon and the lateral border of the supraspinatus foot print. Tendon thickness was measured at the thinnest portion of the tendon (yellow line)

One shoulder specialist resident measured the grade of retraction in mm between the lateral border of the tear and a tangent to the lateral border humeral head footprint in a coronary-t2-weighted image. Tendon thickness was measured at the thinnest portion of the tendon (Fig. 2). Patients were divided into three groups of tendon retraction (A) 0-10 mm, (B) 11-20 mm, and (C) > 20 mm. For tendon thickness 2 groups with (A) > 4 mm and (B) < 4 mm were distinguished.

Patients were divided into 2 groups for BMI: (A) < 25 (normal weight) and (B) < 25 (over weight).

For all groups the mean fat fraction and standard deviation were calculated. Parameters were tested for normal distribution using student’s T Test. Level of significance was calculated using the independent samples Mann-Whitney U-test and the Kruskal-Wallis test. For correlation between fat fraction and different parameters Pearson’s R was calculated. All statistical analyses were performed using SPPS version 14 (IBM, Armonk NY, USA). Differences were considered as statistically significant, when *p* < 0.05. Written informed consent of every patient and institutional ethic committee approval were obtained.

## Results

Twenty-nine of the patients were male and 13 were female. The mean age was 62 years (± 7.7; minimum: 43; maximum: 79 years). The mean fat fraction for all individuals was 19% (± 18). For males the mean fat fraction was 16.2% and for females 25.1% (*p* = 0.23) However, the differences did not reach the level of significance.

Subdivision of tendon retraction into three groups revealed a significant increase of the fat fraction with growing tendon retraction. The mean retraction for all individuals was 15 mm (±14). Individuals with tendons retracted between 0 and 10 mm had a mean fat fraction of 3.7% between 11 and 20 mm 16.7% and greater 20 mm had a mean fat fraction of 37% (*p* = 0.0008).

Tendons thinner than 4 mm had significant higher fat fraction (31.5%) than tendons thicker than 4 mm (9.6%). Duration of symptoms showed significant differences as patients with symptoms longer than 12 months had a mean fat amount of 32.5 and patients with symptoms lesser than 12 months of 8.9% (*p* < 0.0001).

BMI was an independent variable of supraspinatus fat fraction as patients with a BMI < 25 had a mean fat fraction of 15.9% and patients with a BMI > 25 of 17.8% (*p* = 0.78).

Correlation between age and amount of FD was *R* = 0.21 (p0.18).

**Fig. 3:**
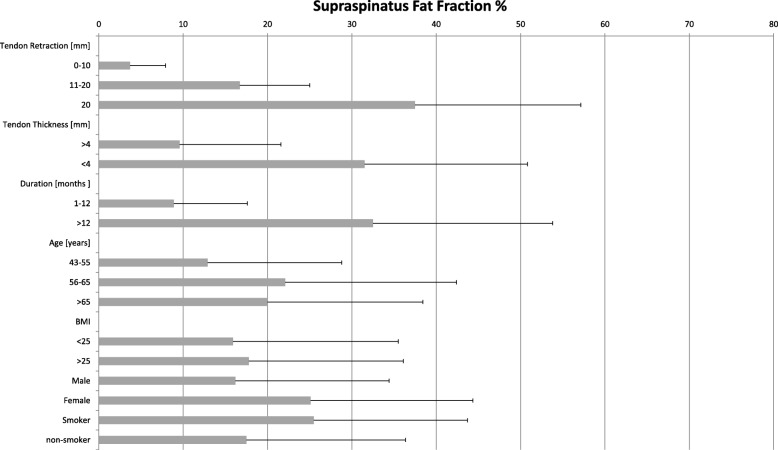
Diagram of mean fat fraction and standard deviation in the supraspinatus muscle belly in different subgroups

Smokers (n=) had a mean fat fraction of 25.5% regarding 17.5% in non-smokers (n) without reaching the level of significance (*p* = 0.64). The patients’ demographic data and results are shown in Tables 1 and 2 (Fig. 3).

**Table 1:** Amount of fat fraction in the supraspinatus muscle belly in the different subgroups

Tendon Retraction [mm]	n	Fat fraction [%]	SD [%]
0–10	15×	3.7	4.2
11–20	13×	16.7	8.2
20	14×	37.5	19.7
Tendon Thickness [mm]			
Larger than 4	24×	9.6	12
Thinner than 4	18×	31.5	19.3
Duration [months]			
1–12	20×	8.9	8.7
> 12	22×	32.5	21.3
Age [years]			
43–55	11×	12.9	15.9
56–65	17×	22.1	20.3
> 65	14×	20	18.4
BMI			
< 25	16×	15.9	19.6
> 25	26×	17.8	18.3
Sex			
Male	11×	16.2	18.2
Female	31×	25.1	19.24
Smoker Status			
Smoker	7×	25,50	18,2
Non-smoker	35×	17,50	18,85

**Table 2:** Patients characteristics

Patients’ features	
Total No. of patients	42
Sex, female: male	29:13
Age [years]	62 (±7.7)
Duration of symptoms [months]	12.3 (±7)
BMI [kg/m^2^]	29 (±4.4)
Tendon retraction [mm]	15.6 (13.7)

## Discussion

Spectroscopic analysis of the fat fraction in full thickness supraspinatus tears revealed positive correlation of tendon retraction and duration of symptoms with the amount of fat in the supraspinatus muscle belly and negative correlation with tendon thickness. Whereas BMI, smoker status and gender seemed to be independent from the intramuscular fat fraction.

Numerous studies reported the impact of these parameters previously, but the majority of these studies relied on the Goutallier scale [5, 19, 20]. Reliability and accuracy of this scale is debatable, as quantitative comparisons revealed a lack of accuracy especially when high amounts of fat occur, as the division between grade 3 and 4 becomes imprecise [8, 16]. The exact pathophysiologic mechanisms of FD are not completely understood and distinction between fatty atrophy and infiltration and vice versa remains imprecise. Scientifically differentiation between these two entities may not be prudent, both may be merged under the term fatty degeneration.

This study found high correlation of tendon retraction with the supraspinatus fat fraction. Tendon retraction is known to strongly correlate with the amount of FD, as it leads to ultrastructural changes in muscle fiber orientation and accumulation of fat cells in the disorientated myofibrillar structure [21, 22]. Different clinical and experimental study designs using the Goutallier method and quantitative methods have revealed that retraction and the amount of fat fraction show high correlation, which has been recently supported by a quantitative study by Lee et al. who found a linear relationship between those variables [14, 16, 21]. Another MRI investigation by Fukuta et al. showed reduction of the cross sectional area of the infraspinatus muscle with progressing retraction [23]. This study furthermore supports these findings, with only very few studies using quantitative methods to this date.

Histological analysis showed that tendon degeneration increases with the progress of tendon disease [24]. Tendon thickness has not been assessed yet in correlation with FD. This study could clearly demonstrate that decrease of tendon thickness correlates with increasing fat fraction.

Symptom duration was also a strong predictor of fatty degeneration in this cohort. This supports the findings of Lee et al., who found a nearly linear relationship in a cohort of 187 patients between symptom duration and the RC fat fraction using a chemical shift MRI [16].

The role of gender to the muscular fat fraction remains controversial, as Barry et al. found that older women with substantial defects more likely develop high fat fractions. Our data indicate higher amount of fat in the female cohort without reaching the level of significance. As only 11 women were included in our study these results may not be representative. Yet, the surgeon should be aware of accelerated development of FD in women.

BMI was independent from intramuscular fat fraction in our cohort. In contrast to our findings Lee et al. found a linear relation between BMI and fat fraction. As increasing BMI has been described to correlate with the fat fraction in the paravertebral muscles its impact to shoulder girdle has not been investigated yet [25]. The difference of the studies remains unclear, but as this study focuses on individuals with full thickness tears, the study of Lee et al. involved healthy and younger individuals. So, the effect of BMI might be attenuated by the other factors with higher impact to the development of FD.

Smokers in this cohort showed higher fat fractions without reaching the level of significance, if smoking has a direct effect to the muscle structure ore promotes FD is not known, but it may represent a less active life style which is related to development of sarcopenia [26].

While this study is an analysis of FD and correlating findings, it may be concluded decreasing tendon diameter as well as increasing retraction may indicate the severity of FD. Although a clinical algorithm cannot be established yet, quantitative analyzation might be able to help identify irreparable RC tears and predict postoperative results after RC repair.

## Conclusion

Detailed understanding of tendon disease needs quantitative methods as the widely used semi-quantitative scales are imprecise and implicate a high amount of guessing. Prospective quantitative assessment of the fat fraction may be a valuable tool to understand the dynamic of fatty degeneration in tendon disease and the effect of RC-repair to its development. The long-distance goal would be identification of a fat fraction threshold when RC repair might be no more beneficial.

## Abbreviations

BMI: Body mass index
FD: Fatty degeneration.
mm: Millimeter.
MRI: Magnet resonance imaging.
RC: Rotator cuff.
SPLASH: Spectroscopic fast low angle shot.
